# Editorial Perspective: Misaligned incentives in mental health research – the case for Registered Reports

**DOI:** 10.1111/jcpp.13898

**Published:** 2023-10-02

**Authors:** Jessie R. Baldwin

**Affiliations:** ^1^ Department of Clinical, Educational and Health Psychology, Division of Psychology and Language Sciences University College London London UK; ^2^ Social, Genetic and Developmental Psychiatry Centre, Institute of Psychiatry, Psychology and Neuroscience King's College London London UK

## Abstract

Current incentive structures reward mental health researchers for producing positive, novel, and clean results. This can promote questionable research practices which contribute to a distorted evidence base, in turn limiting progress in mental health research. Registered Reports (RRs) offer a solution to realign the incentives towards conducting high‐quality, rigorous, and accurate studies, by preventing publication and reporting biases. However, the uptake of RRs in mental health research has so far been limited. This editorial perspective highlights the advantages of RRs for mental health research, before discussing potential challenges and how they can be addressed. Greater uptake of RRs in mental health research could help to promote a fairer research culture, limit publication bias and questionable research practices, and ultimately, improve understanding of mental health.

Current incentive structures are impeding progress in mental health research. Progress relies on researchers reporting accurate results obtained from high‐quality, robust studies. Yet to publish papers and achieve career success, researchers are incentivised by journals, funders, and evaluation committees to produce positive, novel, and clean results. Such incentives can promote questionable research practices (QRPs; such as selective reporting and p‐hacking), which limit research accuracy. As such, researchers' career incentives are misaligned with practices that enable progress in mental health research.

The presence of these incentives in mental health research is demonstrated by a wealth of meta‐scientific evidence. For example, in studies of treatments for depression, 98% of positive antidepressant trials were published, compared to only 48% of negative trials (De Vries et al., 2018). Similarly, in psychotherapy trials, an excess of statistically significant findings was found in published papers (Flint, Cuijpers, Horder, Koole, & Munafò, 2015). Notably, such findings are not only more likely to be published but also cited – with statistically significant findings in psychiatry receiving more than double the citations of nonsignificant studies (Nieminen, Rucker, Miettunen, Carpenter, & Schumacher, 2007).

Unsurprisingly, evidence suggests that these incentives are harming mental health research by promoting QRPs. Indeed, in two surveys (including >7,000 researchers), publication pressure was the strongest predictor of engaging in QRPs (Gopalakrishna et al., 2022; Maggio et al., 2019), independent of other factors, such as career stage and gender. Use of QRPs are not only reported by a high proportion of psychology researchers (e.g. up to 94%; John, Loewenstein, & Prelec, 2012) but are also apparent in the mental health literature. For example, among preregistered clinical trials for psychiatric disorders, more than one in four studies showed evidence of selective outcome reporting (i.e., omitting outcomes with nonsignificant results, or introducing a new, nonregistered primary outcome; Scott, Rucklidge, & Mulder, 2015). Furthermore, in a random sample of studies from psychiatry and psychology journals, 96% of studies reported results which supported the hypothesis, compared to 44% of studies that preregistered their protocols and hypotheses (Scheel, Schijen, & Lakens, 2021), suggesting the presence of selective reporting and/or altering the hypothesis to fit the results (termed ‘HARKing’ – hypothesising after the results are known).

QRPs involving selective reporting and p‐hacking have been found to increase the rate of false positives (Simmons, Nelson, & Simonsohn, 2011) and increase bias in effect sizes (Anderson & Liu, 2023), thereby distorting the evidence base. As such, QRPs are likely to play a role in the low replicability observed in mental health research. For example, only 45% of initial observational studies on psychiatric disorders were replicated by later meta‐analyses (Dumas‐Mallet, Button, Boraud, Munafo, & Gonon, 2016) and only 37% of highly cited findings on psychiatric interventions could be replicated, with initial study effect sizes being overestimated by 132% compared to subsequent studies (Tajika, Ogawa, Takeshima, Hayasaka, & Furukawa, 2015). However, in addition to QRPs, other factors are likely to contribute to low replicability, such as low statistical power, low levels of true effects (Ulrich & Miller, 2020), and heterogeneity in methods.

Clearly, there is a problem, but what can be done to change the incentives and improve the accuracy of mental health research? One solution is to adopt Registered Reports (RRs) – a type of article in which the study protocol is preregistered and peer reviewed prior to research being conducted. If the protocol is considered to be high quality and the topic deemed to be important, the study will be provisionally accepted for publication before data collection and/or analysis commences. After conducting the research, authors submit a complete manuscript for a second stage of peer review. At this stage, reviewers will assess whether the authors have followed the protocol (though post‐hoc analyses are permitted, if clearly labelled) and whether conclusions are justified. The RR format therefore minimises the potential for selective reporting and HARKing in a similar way to pre‐registration (and perhaps more so, as RR reviewers are explicitly required to assess compliance with the Stage 1 protocol). Crucially though, the RR format can uniquely prevent publication bias, as journals commit to publishing studies regardless of the results.

Currently, over 300 journals offer RRs, and over 600 Stage 2 articles (i.e., completed RRs that have undergone both stages of peer review) have been published (Chambers & Tzavella, 2022). In mental health research, several relevant journals offer RRs, such as the *Journal of the American Academy of Child and Adolescent Psychiatry*, *Clinical Psychological Science*, *Child Development*, *Biological Psychiatry: Global Open Science*, and more general journals like *Nature Human Behaviour*, *BMC Medicine*, and *the Royal Society Open Science*.

Published Stage 2 RRs in mental health research have involved both primary and secondary data analysis and have investigated questions such as the impact of covid on mental health (Mansfield et al., 2022), screen use and adolescent depressive symptoms (Ferguson, 2021), and gene–environment correlations and genetic confounding of the relationship between childhood adversity and mental health (Baldwin et al., 2023). Notably though, the overall uptake of RRs in mental health research has so far been limited, with only a handful of published studies.

Why has there been only a limited uptake of RRs in mental health research? It is possible that mental health researchers are not aware of RRs, do not consider them to be advantageous, or believe them to be too challenging. In this editorial, I will address the latter two reasons by considering (a) the advantages of RRs for mental health researchers, and (b) the challenges involved and how they can be mitigated.

## Advantages of RRs

RRs have several advantages for both individual researchers and the wider research field. For individual researchers, there are at least three benefits. First, RRs appear to be less likely to be rejected than typical articles. For example, at *Cortex* (the first journal to introduce RRs), the rejection rate for RRs is 10% at Stage 1 and 0% at Stage 2 (the first and second rounds of peer review, respectively) versus ~90% for regular articles (Chambers & Tzavella, 2022), with similar rates at other journals (e.g. *Royal Society Open Science*). This means that researchers will save time by not having to resubmit to several journals due to unfixable flaws or unappealing results. Second, Stage 1 acceptance of RRs relieves anxiety about difficulties publishing inconvenient results, which is particularly appealing for ECRs, who are first authors on the majority of RRs (Chambers & Tzavella, 2022). Third, RRs reduce pressure to engage in QRPs, allowing researchers to conduct honest and credible research, without limiting their chances of publication.

For the wider research field, initial evidence suggests that RRs can reduce QRPs and improve research quality and reproducibility. Regarding use of QRPs, a study of 127 RRs found that 60% of RRs reported null results, which is five times greater than the rate in regular articles (Allen & Mehler, 2019). Another study found that 56% of RRs reported results that did not support the first hypothesis, compared to 4% of regular psychology/psychiatry articles (Scheel et al., 2021). These findings suggest that RRs can reduce selective reporting and HARKing (although other factors may also contribute to greater levels of null results, such as researchers using RRs to test riskier hypotheses). Importantly though, despite higher levels of null results in RRs, an analysis of 70 RRs found that they are cited a similar amount or more than comparable regular articles (Hummer, Thorn, Nosek, & Errington, 2017). This should reassure authors concerned that RRs could lead to fewer citations due to an increased likelihood of reporting null results, or editors concerned that RRs could reduce journal impact factors.

Evidence also suggests that RRs are perceived to be of higher quality than standard articles. When a sample of RRs from psychology and neuroscience was compared to standard articles matched for topic, author, and journal, 353 scientists rated RRs as being higher in methodological rigour, producing more important discoveries, and having higher overall quality (Soderberg et al., 2021). These results were consistent even among scientists who previously believed that RRs did not improve study quality. RRs may have greater rigour and quality because authors put greater focus on study design to achieve acceptance and reviewers can suggest methodological improvements before the research is conducted, in contrast to standard articles.

Finally, results from RRs have been found to be more computationally reproducible. In a study of 35 RRs with open data and code, 57% were fully computationally reproducible compared to 31% of regular articles (Obels, Lakens, Coles, Gottfried, & Green, 2020). While there is still room for improvement, higher computational reproducibility may be because journals have strict requirements concerning open data and code for RRs, and authors are not incentivised to conceal inconvenient results (Chambers & Tzavella, 2022).

Importantly, the relatively recent introduction of RRs means that meta‐scientific research on the potential advantages of RRs for researchers and the field is not conclusive, and ongoing detailed evaluations are needed. However, the emerging evidence suggests that RRs offer promising advantages in terms of bias control, research quality, and computational reproducibility.

## Challenges of RRs and potential solutions

Despite their many advantages, RRs can also bring the following challenges.

### Time delays

A key challenge of conducting an RR is that typically the Stage 1 peer review takes several months, delaying researchers from starting the research. This can present a barrier for researchers on short‐term contracts and those with little time left on PhDs/grants.

A solution to this problem is for journals to implement a ‘scheduled review’ system to reduce the Stage 1 review time (Chambers & Tzavella, 2022). Under this system, authors first submit a short protocol before writing the Stage 1 manuscript. Editors then invite reviewers to assess a Stage 1 RR manuscript at a fixed date in the future (e.g. 6+ weeks later), with a short time window (e.g. 1 week) for conducting the review. During this time, authors prepare their Stage 1 manuscript, which is reviewed on the scheduled date. This scheduled review enables reviewers to proactively block out time for reviewing in the future, rather than trying to fit a review in alongside other current competing demands.

This scheduled review system is currently offered at Peer Community In Registered Reports (PCI RR; https://rr.peercommunityin.org), a platform that performs peer review of RRs across disciplines, and allows authors to choose the journal it is published in (from a selection) upon acceptance. Journals that subscribe to the PCI RR model (known as PCI RR‐friendly journals) therefore benefit from publishing RRs that have been rigorously peer reviewed, without having to implement their own journal‐based RRs submission workflow. PCI RR‐friendly journals can specify their own additional requirements for acceptance (e.g. in statistical power or bias control), to ensure article quality. Currently, few PCI RR‐friendly journals are focused on mental health (see https://rr.peercommunityin.org/about/pci_rr_friendly_journals), but this is likely to change in the future as the platform grows.

If researchers wish to submit an RR to a journal that is not currently part of the PCI RR model and does not offer a scheduled review, they could take steps to minimise the likelihood of time delays causing problems. For example, if possible, researchers could prioritise submitting RRs early on in contracts and could plan to work on another study while the RR is under review.

### Unforeseen problems preventing adherence to protocol

Another potential challenge of RRs is the risk that unforeseen circumstances could prevent authors from following the Stage 1 protocol. For example, authors may not be able to access a dataset, recruit the necessary sample, or conduct the proposed analysis due to a technical error.

In such an event, authors should explain the situation to the editor and, if possible, propose a contingency plan that allows them to address the research question without inducing additional bias. If the proposed change is major, editors may invite peer reviewers to review the contingency plan prior to authors commencing research. In the worst‐case scenario that a contingency plan is not considered to be sufficient, the authors could withdraw their RR, conduct their study in an alternative way, and publish it as a regular article.

I personally experienced this challenge, as after I received in‐principle Stage 1 acceptance for an RR, I was unable to access a dataset that I had proposed to use. Fortunately though, another dataset was available with similar qualities to the inaccessible dataset. I therefore explained the situation to the editor and wrote a protocol for a contingency plan using the alternative dataset, which was peer reviewed and accepted. My experience highlights the flexibility of editors and reviewers regarding deviations from the protocol, and their acceptance that the scientific process is not always smooth.

### Knowledge of existing datasets

A commonly perceived barrier to RRs is that they are not suitable for research on previously accessed datasets. This is relevant because many mental health researchers analyse the same dataset multiple times, and identifying new, accessible datasets with appropriate data can be challenging. However, conducting an RR on a dataset that has already been accessed could induce bias, as prior knowledge about findings could motivate researchers to pursue certain analyses.

There are three potential solutions to this issue. First, if researchers wish to use a dataset that they have previously accessed, strategies can be implemented to mitigate bias – such as adopting a conservative statistical threshold, applying comprehensive robustness tests, or using multiverse/specification curve analysis techniques (see PCI RR guidance for more information). However, some journals offering RRs do not permit analysis of already accessed datasets, so researchers should check their target journal guidelines before submitting. Second, researchers could consider using nonaccessed data from a previously accessed dataset. For example, if there has been a new wave of data collection, researchers could propose to use outcome measures from the new data. Third, if possible, researchers could propose to use a new dataset which they have not accessed before – using resources such as the Catalogue for Mental Health Measures (https://www.cataloguementalhealth.ac.uk) or Landscaping Longitudinal Datasets (https://www.landscaping‐longitudinal‐research.com) to identify relevant samples. Researchers can then provide proof that they have not accessed the dataset (or key variables) through a letter from the data provider or self‐certification.

### Statistical power requirements

A final potential barrier is that certain journals require very high statistical power for RRs (e.g. ≥0.90 or 0.95 in *Nature Human Behaviour* or *BMC Medicine*). While this helps to ensure methodological rigour, it can be challenging to obtain very high power when studying small effect sizes that are common in mental health or hard‐to‐reach samples. However, researchers should not be deterred from considering an RR if they do not have very high power, as a number of relevant mental health journals have no requirements for power (e.g. *JAACAP, Clinical Psychological Science*) or lower requirements (e.g. 0.80 power in *Child Development*).

## Conclusion

RRs shift the incentives away from producing positive, novel, and clean results (at the cost of accuracy) towards conducting accurate, high‐quality, and rigorous research. However, the uptake of RRs in mental health research has so far been limited. This may be due to a lack of awareness of RRs and their benefits, or consideration of the challenges involved in conducting an RR. Nevertheless, initial evidence suggests that RRs could not only reduce bias and improve the quality of mental health research but also benefit researchers by preventing results‐based rejections. Moreover, the challenges involved in RRs can be addressed by journals taking steps to be efficient and flexible, and/or authors taking steps to minimise bias, promote transparency, and plan effectively. Of course, RRs are not a panacea for research quality, and other reforms are also needed to improve mental health research (e.g. greater statistical power, more diverse and inclusive samples, improved measurement, and better causal inference). However, by limiting publication bias and QRPs in mental health research, RRs can help make an important contribution to progressing understanding of mental health.

## Data Availability

Data sharing not applicable to this article as no datasets were generated or analysed during the current study.
